# Latent HIV-1 is activated by exosomes from cells infected with either replication-competent or defective HIV-1

**DOI:** 10.1186/s12977-015-0216-y

**Published:** 2015-10-26

**Authors:** Claudia Arenaccio, Simona Anticoli, Francesco Manfredi, Chiara Chiozzini, Eleonora Olivetta, Maurizio Federico

**Affiliations:** National AIDS Center, Istituto Superiore di Sanità, Viale Regina Elena, 299, 00161 Rome, Italy; Department of Sciences, University Roma Tre, Rome, Italy

**Keywords:** Exosomes, HIV-1 latency, CD4^+^ T lymphocytes, Nef, ADAM17

## Abstract

**Background:**

Completion of HIV life cycle in CD4^+^ T lymphocytes needs cell activation. We recently reported that treatment of resting CD4^+^ T lymphocytes with exosomes produced by HIV-1 infected cells induces cell activation and susceptibility to HIV replication. Here, we present data regarding the effects of these exosomes on cells latently infected with HIV-1.

**Results:**

HIV-1 latently infecting U937-derived U1 cells was activated upon challenge with exosomes purified from the supernatant of U937 cells chronically infected with HIV-1. This effect was no more detectable when exosomes from cells infected with HIV-1 strains either *nef*-deleted or expressing a functionally defective Nef were used, indicating that Nef is the viral determinant of exosome-induced HIV-1 activation. Treatment with either TAPI-2, i.e., a specific inhibitor of the pro-TNFα-processing ADAM17 enzyme, or anti-TNFα Abs abolished HIV-1 activation. Hence, similar to what previously demonstrated for the exosome-mediated activation of uninfected CD4^+^ T lymphocytes, the Nef-ADAM17-TNFα axis is part of the mechanism of latent HIV-1 activation. It is noteworthy that these observations have been reproduced using: (1) primary CD4^+^ T lymphocytes latently infected with HIV-1; (2) exosomes from both primary CD4^+^ T lymphocytes and macrophages acutely infected with HIV-1; (3) co-cultures of HIV-1 acutely infected CD4^+^ T lymphocytes and autologous lymphocytes latently infected with HIV-1, and (4) exosomes from cells expressing a defective HIV-1.

**Conclusions:**

Our results strongly suggest that latent HIV-1 can be activated by TNFα released by cells upon ingestion of exosomes released by infected cells, and that this effect depends on the activity of exosome-associated ADAM17. These pieces of evidence shed new light on the mechanism of HIV reactivation in latent reservoirs, and might also be relevant to design new therapeutic interventions focused on HIV eradication.

## Background

HIV infection is efficiently counteracted by combination anti-retroviral therapy (cART) which, despite preventing disease progression, does not eradicate virus infection which persists in a latent form. The latent reservoir is generated by virus entry in activated CD4^+^ T lymphocytes committed to return to a resting state [1], even though also resting CD4^+^ T lymphocytes can be latently infected [2]. Thus, one may assume that HIV reservoir is prevalently composed by memory CD4^+^ T lymphocytes. Therapy interruption leads to viral rebound, which has been observed also after a long time from cART discontinuation [3]. This event is most likely the consequence of the extended half-life of HIV reservoir (about 44 months) [4], which can also expand through homeostatic proliferation [5], i.e. an IL-7-dependent mechanism allowing central memory CD4^+^ T lymphocytes to proliferate without cell differentiation/activation.

HIV-1 infected cells release nanovesicles in the form of viral particles and non-viral vesicles including exosomes. The latter are lipid bilayer vesicles of 50–100 nm, which form intracellularly upon inward invagination of endosome membranes [6]. These intraluminal vesicles become part of multivesicular bodies (MVBs) and either undergo lysosomal degradation, or are released into extra-cellular space upon fusion of MVBs with plasma membrane. Nanovesicles similar to exosomes can be released also through direct extrusion of plasma membrane [7]. In some instances methods of purification and analysis cannot distinguish between endosome-produced nanovesicles and vesicles of similar dimensions but released from plasma membrane. For the sake of clarity, these nanovesicles are here defined as exosomes irrespectively of their biogenesis.

Budding of HIV and related lenti- and retroviruses is preceded by interaction with a number of cell factors involved in exosome biogenesis such as Alix, Tsg101, and several other components of the endosomal sorting complex required for transport (ESCRT) [8]. The convergence of exosome and HIV biogenesis implies the possibility that viral products can be uploaded in exosomes as observed in the case of HIV-1 Gag [9] and Nef [10, 11].

Exosomes are part of the intercellular communication network [12]. They incorporate messenger RNAs, microRNAs, and proteins which can be functional in target cells [13]. These evidences posed the question over whether or not biologically active molecules uploaded in exosomes from HIV infected cells can influence intercellular communication, and the possible relevance of this event in HIV pathogenesis. On this subject, we recently provided evidence that primary CD4^+^ T lymphocytes infected with HIV-1 release exosomes which activate quiescent human primary CD4^+^ T lymphocytes which in turn become permissive to HIV-1 infection [14, 15]. The key event for the activation of resting lymphocytes is the uploading of activated ADAM17 in exosomes released by infected cells. ADAM17 belongs to the family of ADAM (a disintegrin and metalloprotease) enzymes [16]. It is a multi-domain, transmembrane, Zn^2+^-dependent proteinase whose inactive form is cleaved by furin in the *trans*-Golgi network. The most studied function of ADAM17 (also known as TACE, TNFα-converting enzyme) is the processing of pro-TNFα into its active form. Considering that TNFα is a pleiotropic cytokine, uploading of activated ADAM17 in exosomes could have broad consequences in many aspects of HIV-induced pathogenesis.

Here, we provide evidence that exosomes from cells expressing either replication-competent or non-producer HIV-1 activate latent HIV-1 through a mechanism depending on both ADAM17 and TNFα.

## Results

### Purification of exosomes from U937 cell lines chronically infected with HIV-1

**Fig. 1:**
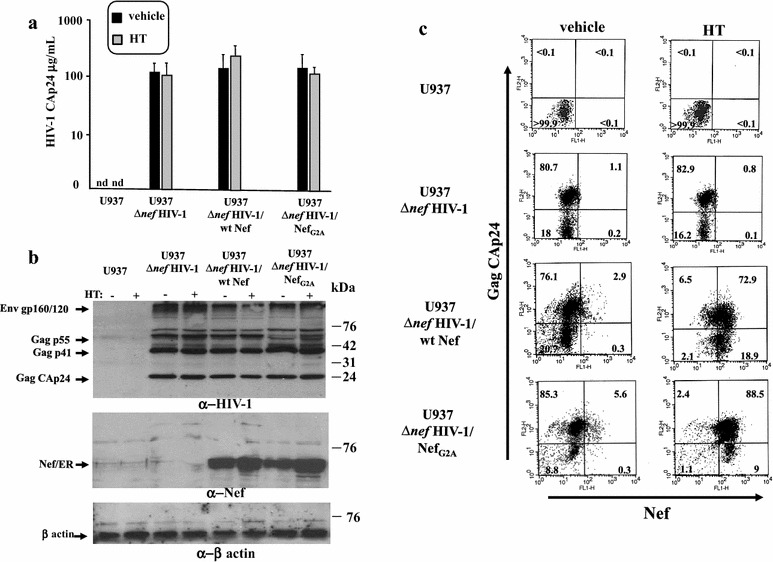
Characterization of HIV-1 chronically infected U937-based cell lines expressing Nef in a regulatable way. **a** Determination of viral release from HIV-1 chronically infected U937-based cell lines by HIV-1 CAp24 ELISA. Cultures of 10^6^ cells/mL of U937 cells either uninfected or chronically infected with Δ*nef* HIV-1, the latter stably transfected with vectors expressing either ER alone (U937 Δ*nef* HIV-1), ER fused with wt Nef (U937 Δ*nef* HIV-1/wt Nef), or ER fused with the Nef G2A mutant (U937 Δ*nef* HIV-1/Nef_G2A_), were carried out for 48 h with either HT or equal volume of vehicle. Afterwards, supernatants were harvested, clarified, and viral contents measured in terms of concentration of CAp24. Shown are the mean values +SD as calculated from duplicate conditions run in seven independent experiments. Nd: not detectable. **b** Western blot analysis for expression of HIV-1 related products in the HIV-1 chronically infected U937 cell lines either untreated (−) or treated (+) with HT for 48 h. Cell lysates from the U937-based cell lines were resolved in 12 % SDS-PAGE and probed for Gag, Env (*upper panel*), and Nef expression (*middle panel*). Signals were normalized by β-actin detection (*lower panel*). On the *right* of each *panel*, molecular weight markers are given in kilodaltons (kDa). On the *left*, migrations of relevant products are indicated. The results are representative of five independent experiments. **c** FACS analysis for the expression of both Gag and Nef products. The different U937-based cell lines were incubated for 48 h with HT or equal volume of vehicle, then permeabilized and labeled with both anti-Gag and—Nef mAbs. Percentages of events are reported in the respective quadrants. The results are representative of two independent experiments

Exosomes produced by HIV-1 infected cells can induce cell activation in target CD4^+^ T lymphocytes [14, 15]. Since we proved that TNFα is part of the underlying mechanism, we were interested in assessing possible effects of exosomes released by HIV-1 infected cells on latent HIV-1, which has been reported to be activated by this cytokine [17]. To this end, the availability of large amounts of pure exosomes was mandatory. Most conveniently, they can be obtained from supernatants of cell lines stably expressing wild-type HIV-1. However, basically all HIV-1 chronically infected cell lines so far isolated express HIV-1 genomes defective for one or more genes. In particular, the anti-cellular effects induced by the stable expression of Nef [18–20] hamper the isolation of cell lines stably infected with Nef-expressing HIV-1. To overcome this hindrance, we isolated HIV-1 chronically infected cells starting from the previously described U937 cell lines stably transfected with either wild-type (wt) HIV-1 Nef or mutants thereof fused at their C-terminus with the estrogen receptor (ER) [21]. The ER domain keeps Nef in an inactive state through a steric hindrance which is relieved upon binding with the estrogen antagonist 4-hydroxytamoxifen (HT). In detail, U937 cells stably transfected with vectors expressing ER either alone, fused with wt Nef, or with the Nef_G2A_ mutant defective for ADAM17 uploading in exosomes [14], were infected with Δ*nef*NL4-3 HIV-1. Infected cell cultures were carried out in the absence of HT until the percentage of infected cells was >75 %, which remained stable over time. The amount of HIV-1 particles released by the three cell lines did not change significantly after HT stimulation (Fig. 1a). Consistently, no apparent variations in the expression of HIV-1 products were seen after HT treatment (Fig. 1b), except for a slight increase of Nef-ER possibly due to an improved stability of the fusion products [22]. As assessed by intracytoplasmic FACS analysis, the striking majority of both U937 Δ*nef* HIV-1/wt Nef and U937 Δ*nef* HIV-1/Nef_G2A_ cells co-expressed both Gag and Nef products after HT treatment (Fig. 1c).

**Fig. 2:**
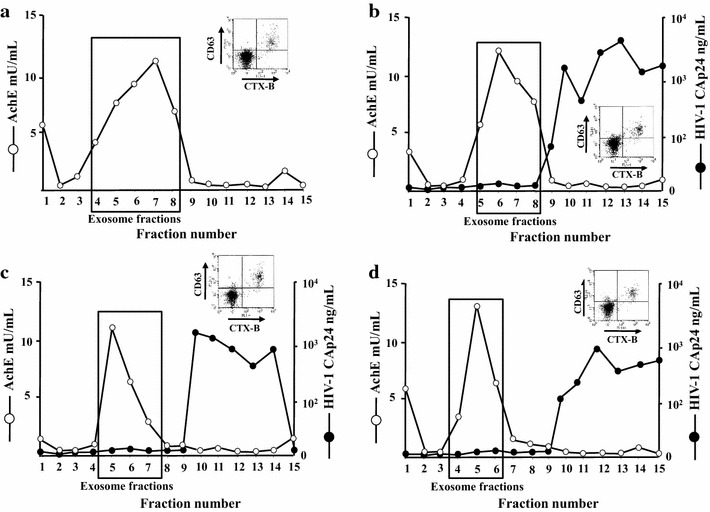
Purification and characterization of exosomes from parental and HIV-1 chronically infected U937 cells expressing Nef in a regulatable way. AchE activity and, for HIV-1 infected cells only, HIV-1 Gag CAp24 contents were measured in fractions from 6 to 18 % iodixanol gradients loaded with vesicles obtained by differential centrifugations of supernatants from **a** U937, **b** U937 Δ*nef*HIV-1, **c** U937 Δ*nef*HIV-1/wtNef, and **d** U937 Δ*nef*HIV-1/Nef_G2A_ cells after treatment with HT for 48 h. In the insets, shown are FACS analyses for the presence of both GM1 and CD63 in nanovesicles recovered from pools of AchE positive fractions of iodixanol gradients. Vesicles were bound to aldehyde/sulfate latex beads and then labeled with FITC-conjugated CTX-B and PE-conjugated anti-CD63 mAb. The results are representative of two independent experiments

For exosome purification, parental U937 as well as the different HIV-1 chronically infected cell lines were treated with HT and, 48 h later, supernatants were harvested and processed by differential centrifugations. The resulting nanovesicle pellets were then loaded on 6–18 % discontinuous iodixanol gradients to separate exosomes from HIV-1 particles. Gradient fractions were then assayed in terms of acetylcholinesterase (AchE) activity (i.e., a classical exosome marker) [23] for exosome detection, and, whenever relevant, HIV-1 Gag products (Fig. 2). Nanovesicle preparations recovered from AchE strongly positive fractions were further characterized in terms of presence of both CD63 (i.e., a tetraspanin typically associated with exosomes) [24], and monosialotetrahexosylganglioside (GM1), i.e., a component of nanovesicle-associated lipid rafts detectable through binding with the subunit B of cholera toxin (CTX-B) (Fig. 2, insets) [25].

### HIV-1 latently infecting U1 cells is activated by exosomes from HIV-1 infected cells

We investigated possible virologic effects of exosomes from HIV-1 infected cells on HIV-1 latently infected cells. In a first instance, we analyzed the effects on U1 cells [26], i.e., U937-derived cells with two inactive HIV-1 proviruses integrated which express mutated/inactive *tat*. In particular, *tat* from one HIV-1 provirus lacks the ATG start codon, whereas the other expresses a Tat protein whose functions are heavily compromised by the H to L substitution at the amino acid 13 [27]. Treatment of U1 cells with either wild-type Tat, tumor necrosis factor (TNF)α, phorbol myristate acetate (PMA), or phytohemagglutinin (PHA) results in virus activation [26–28].

**Fig. 3:**
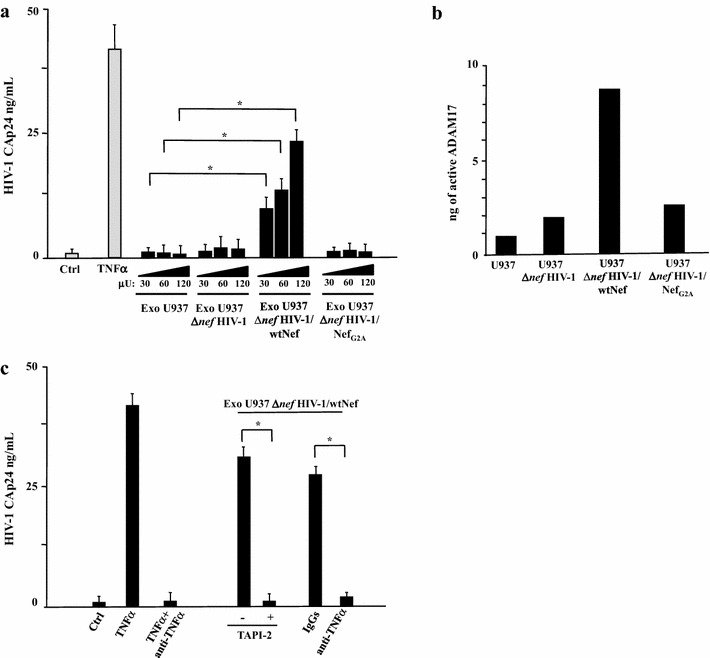
HIV-1 latently infecting U1 cells is activated upon challenge with exosomes from HIV-1 infected cells in a Nef-, TNFα-, and ADAM17-dependent manner. **a** Different amounts of exosomes (i.e., from 30 to 120 μU of AchE activity) purified from supernatants of the indicated cell lines were used to challenge 5 × 10^4^ U1 cells in U-bottom 96 well plates. After 24 h, the cells were extensively washed, and the amount of released HIV-1 was measured in terms of CAp24 concentration in the supernatants, after additional 24 h. As a control, cells were left untreated (Ctrl) or treated with 100 ng/mL of recombinant TNFα. The results are the mean values + SD from five independent experiments carried out with duplicates. *p < 0.05. **b** ADAM17 activity detected in 1 mU of exosomes purified from the supernatants of the indicated U937-derived cell lines. Shown are mean amounts of active ADAM17 + SD detected in quadruplicate samples from a representative exosome preparation from each cell line. **c** Effects of TAPI-2 and anti-TNFα on the activation of latent HIV-1 induced by exosomes from HIV-1 expressing cells. U1 cells (5 × 10^4^/condition) were challenged with 100 μU of exosomes from HIV-1 infected cells expressing wt Nef. Then, cells were left untreated or incubated in the presence of either 1 μM TAPI-2, 160 ng/mL of anti-TNFα neutralizing antibodies, or equivalent amounts of unrelated, isotype-specific IgGs. As a control, cells were treated with 100 ng/mL of recombinant TNFα in the presence or not of the anti-TNFα neutralizing antibodies. After 24 h, cells from all conditions were extensively washed, re-seeded in the appropriate conditions, and the amount of HIV-1 released in supernatants was measured as CAp24 concentration after additional 24 h. The results are the mean values + SD from three independent experiments carried out with duplicates. *p < 0.05

We treated U1 cells with different amounts (i.e., from 30 to 120 μU of AchE activity) of exosomes purified from HT-treated U937 cells expressing either ER alone, both Δ*nef* HIV-1 and ER, Δ*nef* HIV-1 and wtNef-ER, or Δ*nef* HIV-1 and Nef_G2A_-ER. Only the challenge with exosomes from HIV-1 infected cells expressing wt Nef induced activation of latent HIV-1 (Fig. 3a). The effect appeared to be dose-dependent, and required the expression of a functional Nef in exosome-producing cells.

Nef induces exosome uploading of activated ADAM17, which, once ingested by target cells, leads to the release of mature TNFα [14, 15, 29]. To assess whether a similar mechanism was at the basis of the exosome-dependent activation of latent HIV-1 in U1 cells, we first measured the amount of activated ADAM17 in exosome preparations. As expected, exosomes produced by cells expressing wtNef associated with much higher amounts of activated ADAM17 than those from the other cell lines assayed (Fig. 3b). Afterwards, either TAPI-2 (i.e., a specific inhibitor of ADAM17) [30] or neutralizing anti-TNFα Abs were added to U1 cell cultures immediately after exosome challenge. Both treatments led to a sharp decrease of the exosome-induced activation of latent HIV-1 (Fig. 3c), thus strongly suggesting that the phenomenon we observed was driven by a mechanism similar to that previously described for uninfected quiescent CD4^+^ T lymphocytes [14, 15]. Of importance, all exosome preparations we used were found devoid of detectable amounts of TNFα (not shown).

Together, these results represent a preliminary indication that latent HIV-1 infection can be activated by exosomes from HIV-1 productively infected cells.

### Set up of a system of HIV-1 latent infection in unstimulated primary CD4^+^ T lymphocytes

**Fig. 4:**
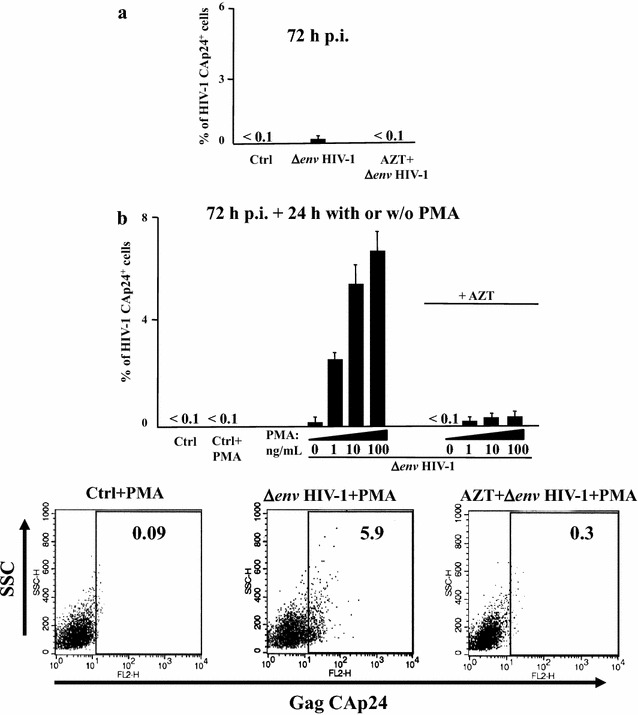
Set up of a system of HIV-1 latent infection. Untouched CD4^+^ T lymphocytes were purified from PBMCs of healthy donors and challenged by spinoculation with (VSV-G) Δ*env* HIV-1 in the presence or not of AZT. As a control, conditions with unchallenged CD4^+^ T lymphocytes (Ctrl) were also run. After 48 h, cells were extensively washed and then left in culture for additional 24 h. **a** Intracytoplasmic CAp24 FACS analysis of CD4^+^ T lymphocytes 72 h post-infection. The results are the mean values + SD from nine independent experiments carried out with duplicates. **b** Intracytoplasmic CAp24 FACS analysis of CD4^+^ T lymphocytes which, 72 h post-infection, were either activated for 24 h by the indicated doses of PMA+ 0.5 μg/μL of ionomycin (PMA) or left untreated. Ctrl: uninfected CD4^+^ T lymphocytes. The results are the mean values + SD from three independent experiments carried out with duplicates. In the bottom panels, shown are representative raw results obtained by the intracytoplasmic CAp24 FACS analysis of CD4^+^ T lymphocytes either uninfected (Ctrl), or latently infected with (VSV-G) Δ*env* HIV-1 in the presence or not of AZT, and treated for 24 h with PMA + ionomycin

Next, we tried to extend our results to a more physiologic context of HIV-1 latent infection. Different protocols aimed at recovering HIV-1 latently infected primary cells have been described [31–37], and recently compared [38]. We practiced the method already described by Greene and coll. [31] with few modifications. In detail, CD4^+^ T lymphocytes were isolated from peripheral blood mononuclear cells (PBMCs) of healthy donors by negative immunomagnetic selection. Untouched cells were challenged by spinoculation with Δ*env*HIV-1 pseudotyped with vesicular stomatitis virus (VSV)-G protein in the presence or not of 10 μM azidothymidine (AZT), and 48 h later extensively washed. We observed that after additional 24 h viral replication remained basically at background levels, as determined by the assessment of CAp24 in both cells (Fig. 4a) and supernatants (not shown). Treatment of challenged cultures with different amounts of PMA led to the appearance of 3–8 % of cells expressing HIV-1 within 24 h (Fig. 4b). The percentage of infected cells then decreased (not shown) most likely as a consequence of the cytotoxic effect of HIV-1 which, in view of its *env* defectiveness, cannot spread in bystander activated cells.

HIV-1 activation did not take place in PMA stimulated CD4^+^ T lymphocytes treated with AZT before infection (Fig. 4b), demonstrating that the results from FACS analysis were not biased by carryover from the viral input.

### Exosomes from HIV-1 infected cells activate latent HIV-1 in primary CD4^+^ T lymphocytes

**Fig. 5:**
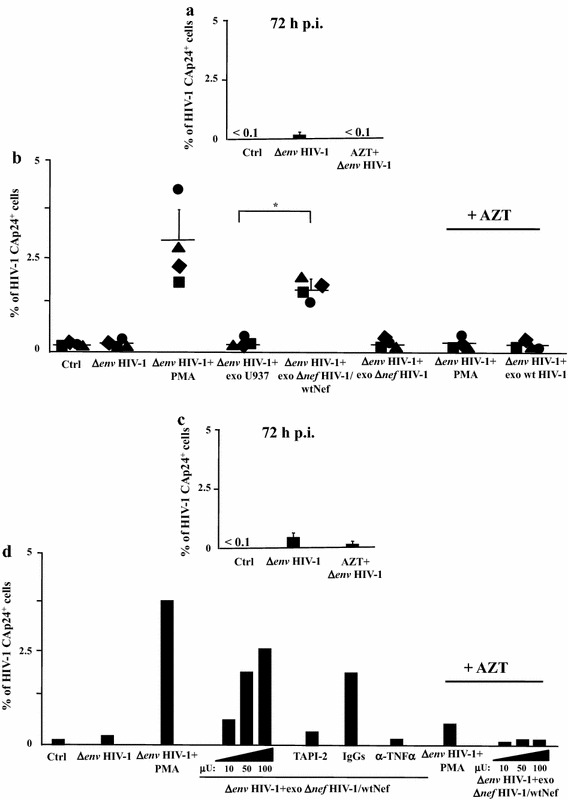
HIV-1 latently infecting primary CD4^+^ T lymphocytes is activated upon challenge with exosomes from HIV-1 infected cells. Untouched CD4^+^ T lymphocytes isolated from PBMCs of four healthy donors were challenged by spinoculation with (VSV-G) Δ*env*HIV-1 in the presence or not of AZT. As control, conditions with unchallenged CD4^+^ T lymphocytes (Ctrl) were included. After 48 h, cells were extensively washed and then left in culture for additional 24 h. **a** Intracytoplasmic CAp24 FACS analysis of CD4^+^ T lymphocytes 72 h post-infection. The results are the mean values + SD calculated after challenge of CD4^+^ T lymphocytes from four healthy donors in duplicate conditions. **b** Intracytoplasmic CAp24 FACS analysis of HIV-1 latently infected CD4^+^ T lymphocytes 24 h after spinoculation with 100 μU of exosomes purified from either uninfected U937 cells or their counterpart chronically infected with Δ*nef*HIV-1 and expressing or not wt Nef. As control, cells were treated with 10 ng/mL of PMA+ 0.5 μg/μL of ionomycin (PMA) or left untreated (Ctrl). In addition, CD4^+^ T lymphocytes originally challenged with (VSV-G) Δ*env*HIV-1 in the presence of AZT were treated with either PMA + ionomycin or exosomes from HIV-1 infected U937 cells expressing wt Nef. Shown are the results calculated as mean percentage values of triplicate cultures of CD4^+^ T lymphocytes from each donor. The inter-donor mean values + SD are also presented. *p < 0.05. The same experiments whose data are shown on panels **a** and **b** have been reproduced using PBMCs from two healthy donors and three doses (from 10 to 100 μU) of exosomes from cells infected with Δ*nef*HIV-1 and expressing wt Nef. In addition, CD4^+^ T quiescent lymphocytes challenged with 100 μU of exosomes were treated with either TAPI-2, anti-TNFα Abs, or isotype matched IgGs soon after the exosome challenge. **c** Intracytoplasmic CAp24 FACS analysis of CD4^+^ T lymphocytes 72 h post-infection. The results are the mean values + SD calculated after challenge of CD4^+^ T lymphocytes from two healthy donors in duplicate conditions. **d** Intracytoplasmic CAp24 FACS analysis of HIV-1 latently infected CD4^+^ T lymphocytes 24 h after spinoculation with the exosomes. As control, cells were treated with 10 ng/mL of PMA+ 0.5 μg/μL of ionomycin (PMA) or left untreated (Ctrl). In addition, CD4^+^ T lymphocytes originally challenged with (VSV-G) Δ*env*HIV-1 in the presence of AZT were treated with either PMA+ ionomycin or exosomes. Shown are the results calculated as mean percentage values of duplicate cultures of CD4^+^ T lymphocytes from each donor

The here above described experimental system was instrumental to determine possible effects of exosomes from HIV-1 infected cells on HIV-1 latently infecting primary lymphocytes. To this aim, untouched CD4^+^ T lymphocytes from four different healthy donors were infected with (VSV-G) Δ*env*HIV-1 in the presence or not of AZT. After 3 days, the percentage of cells actively expressing HIV-1 was assessed by intracellular Gag-specific FACS analysis (Fig. 5a). At this time, the absence of detectable Gag-expressing cells in the infected cell population was a mandatory condition to proceed with the exosome challenge. Purified exosomes (100 μU) from U937, U937 Δ*nef* HIV-1 and U937 Δ*nef* HIV-1/wtNef cells were used to challenge the cultures of HIV-1 latently infected CD4^+^ T lymphocytes, whose percentages of HIV-1 expressing cells were measured 24 h later. As shown in Fig. 5b, CD4^+^ T lymphocytes expressing Gag proteins were detectable only after challenge with exosomes from Nef expressing HIV-1 infected cells. As expected, no Gag-positive CD4^+^ T lymphocytes were detected when cells were treated with AZT at the time of HIV-1 challenge. The efficiency of activation of latent HIV-1 depended on the amount of exosomes used, and appeared strongly reduced by treatment with either TAPI-2 or anti-TNFα Abs at the time of exosome challenge (Fig. 5c, d).

These data which were obtained using a system of HIV-1 latency based on primary lymphocytes appeared fully consistent with the results we had previously obtained with U1 cells.

### Activation of latent HIV-1 in primary CD4^+^ T lymphocytes challenged with exosomes purified from HIV-1 infected primary cells

**Fig. 6:**
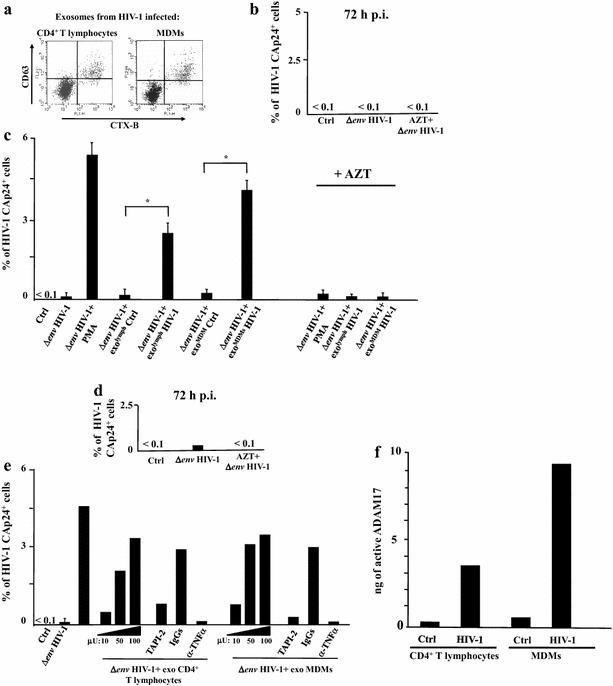
Exosomes from HIV-1 productively infected primary cells activates latent HIV-1 infecting primary CD4^+^ T lymphocytes. Untouched CD4^+^ T lymphocytes from PBMCs of healthy donors were challenged by spinoculation with (VSV-G) Δ*env*HIV-1 in the presence or not of AZT. As a control, conditions with unchallenged CD4^+^ T lymphocytes (Ctrl) were included. After 48 h, cells were extensively washed, and then left in culture for additional 24 h. Afterwards, exosome challenge was performed as described below. **a** FACS analysis for the presence of both GM1 and CD63 in nanovesicles recovered from pools of AchE positive fractions of iodixanol gradients loaded with nanovesicle pellets from supernatants of either CD4^+^ T lymphocytes or MDMs infected with (VSV-G) wtHIV-1. Vesicles were bound to aldehyde/sulfate latex beads and then labeled with FITC-conjugated CTX-B and PE-conjugated anti-CD63 mAb. **b** Intracytoplasmic CAp24 FACS analysis of CD4^+^ T lymphocytes 72 h post-infection. The results are expressed as mean HIV-1 Gag^+^ percentage calculated after challenge of CD4^+^ T lymphocytes from three healthy donors analyzed in duplicate conditions. **c** Intracytoplasmic CAp24 FACS analysis of HIV-1 latently infected CD4^+^ T lymphocytes 24 h after spinoculation with 100 μU of exosomes purified from HIV-1 infected CD4^+^ T lymphocytes or MDMs. As a control, the cells were challenged with equivalent amounts of exosomes from uninfected CD4^+^ T lymphocytes or MDMs. CD4^+^ T lymphocytes were also treated with PMA + ionomycin (PMA) or left untreated (Ctrl). In addition, CD4^+^ T lymphocytes originally challenged with (VSV-G) Δ*env*HIV-1 in the presence of AZT were treated with either PMA + ionomycin or exosomes from HIV-1 infected cells, i.e. CD4^+^ T lymphocytes and MDMs. The results are reported as mean percentage values + SD of duplicate cultures of CD4^+^ T lymphocytes from three healthy donors. *p < 0.05. The same experiment whose results are shown in the panel C has been conducted with CD4^+^ T lymphocytes from two healthy donors which were latently infected with Δ*env*HIV-1 in the presence or not of AZT. Panel **d** reports the mean percentage values of HIV-1 expressing cells 72 h after challenge. Then, cells were challenged with three doses of each exosome preparation, i.e., from 10 to 100 μU, or with PMA as a control. In addition, cell challenged with 100 μU of exosomes were treated with either TAPI-2, anti-TNFα Abs, or isotype matched IgGs soon after exosome challenge. The results calculated as mean percentage values of duplicate cultures of CD4^+^ T lymphocytes from each donor are shown in *panel* **e**, **f**. ADAM17 activity detected in 1 mU of exosomes purified from the supernatants of both uninfected or HIV-1 infected CD4^+^ T lymphocytes and MDMs. Shown are mean values of ng of active ADAM17 detected in quadruplicate samples from a representative exosome preparation from each cell culture

To add further significance to our observations, we reproduced the experiments on HIV-1 latently infected CD4^+^ T lymphocytes using exosomes purified from primary cells (i.e., CD4^+^ T lymphocytes and monocyte-derived macrophages, MDMs) acutely infected with HIV-1. In detail, both PHA-stimulated CD4^+^ T lymphocytes and MDMs were infected with (VSV-G) HIV-1, and, when >50 % of cells expressed HIV-1, supernatants were harvested and exosomes purified through differential centrifugations. Nanovesicles were loaded on a 6–18 % discontinuous gradient and, as here above described, those comprised in AchE positive fractions were formally identified as exosomes on the basis of the binding with CTX-B and the presence of CD63 (Fig. 6a).

HIV-1 latently infected CD4^+^ T lymphocytes were recovered 72 h after virus challenge (Fig. 6b) as described above, and treated with 100 μU of exosomes purified from either uninfected or HIV-1 productively infected CD4^+^ T lymphocytes and MDMs. We observed HIV-1 activation within latently infected cells challenged with exosomes isolated from infected but not uninfected primary cell cultures. Virus activation was no more detectable when target cells were pre-treated with AZT (Fig. 6c), its efficiency depended on the size of the exosome input, and was basically abolished by the treatment with either TAPI-2 or anti-TNFα (Fig. 6d, e). The latter results appeared consistent with the detection of activated ADAM17 in exosome preparations from infected but not uninfected CD4^+^ T lymphocytes and MDMs (Fig. 6f).

We concluded that exosomes from HIV-1 infected primary cells can activate latent HIV-1.

### Activation of latent HIV-1 in primary CD4^+^ T lymphocytes upon co-cultivation with autologous CD4^+^ T lymphocytes productively infected with HIV-1

Transfer of exosomes from HIV-1 infected cells to HIV-1 latent reservoir is expected to be accompanied by transmission of additional nano- and microvesicles (including HIV-1 particles), as well as soluble factors. To better evaluate the potential role of additional extra-cellular factors in the exosome-induced activation of latent HIV-1, we set up *trans*-well co-cultures of HIV-1 productively infected cells with HIV-1 latently infected CD4^+^ T lymphocytes. In detail, CD4^+^ T lymphocytes were infected with (VSV-G) HIV-1 2 days after PHA-stimulation, and, at the peak of HIV-1 replication, put in the upper chamber of a *trans*-well plate in the absence of exogenous cell activation factors, such as PHA, IL-2. In the lower chamber, autologous HIV-1 latently infected CD4^+^ T lymphocytes were seeded. Controls in the upper chamber included uninfected activated CD4^+^ T lymphocytes, whereas in the lower chamber comprehended resting CD4^+^ T lymphocytes either uninfected or treated with AZT at the time of infection. After 48 h of co-cultivation in the presence of AZT, HIV-1 activation in latently infected CD4^+^ T lymphocytes was evaluated by intracellular HIV-1 Gag FACS analysis. In these co-culture experiments the effects of two inhibitors of exosome biogenesis (i.e., GW4869 and spiroepoxide) [39–43], TAPI-2, and anti-TNFα Abs, as well as the role of Nef expression in HIV-1 productively infected cells were evaluated. Our data show that (Table 1): (1) activation of latent HIV-1 did occur in co-cultures comprising CD4^+^ T lymphocytes productively infected with wtHIV-1; (2) activation was no more detectable in the presence of the exosome inhibitors; (3) treatment with either TAPI-2 or anti-TNFα Abs effectively reduced the number of HIV-1-expressing cells in latently infected CD4^+^ T lymphocytes, and (4) Nef expression in productively infected cells was mandatory for optimal activation of latent HIV-1. Hence, as previously observed with purified exosomes, also in the co-culture system exosomes from HIV-1 infected cells induced activation of latent HIV-1 through a Nef- and ADAM17-dependent way, whereas the presence of additional extra-cellular factors appeared ineffective. As expected, in the presence of AZT no HIV-1 infected cells were found when uninfected, resting CD4^+^ T lymphocytes were used as target cells, thus excluding events of productive infection due to transmission of HIV-1 from productively infected cells. Also, transmission of cell-activating soluble factors possibly released by productively infected and/or activated cells did not seem to interfere with the results we obtained since no or very little HIV-1 activation was seen: (1) in latently infected cells co-cultivated with uninfected, activated cells; and (2) in co-cultures carried out in the presence of either the exosome inhibitors, TAPI-2, or anti-TNFα Abs, and (3) when effector cells were infected with Δ*nef* HIV-1. Finally, as observed in our previous experiments, the lack of HIV-1 activation in unstimulated CD4^+^ T lymphocytes treated with AZT before infection and co-cultivated with HIV-1 expressing cells indicated that the Gag-specific FACS assay detected de novo produced intracellular Gag molecules only.

**Table 1:** HIV-1 latently infecting primary CD4^+^ T lymphocytes is activated upon co-culture with HIV-1 infected autologous lymphocytes in both ADAM17- and Nef-dependent ways

No. of donors assayed	Effector cells in upper chambers	Effector cells: % of CAp24^+^ cells 3 days p.i.	Co-culture conditions	Target cells in lower chambers: % of CAp24^+^ cells after 2 days of co-culture		
				Mock	ΔenvHIV-1	ΔenvHIV-1 +AZT
5	Ctrl	<0.1	Complete medium	<0.1	<0.1	<0.1
5	wtHIV-1	31.2	Complete medium	<0.1	8.7	<0.1
5	wtHIV-1	32	GW4869+ Spiroepoxide	<0.1	1.6	0.4
3	wtHIV-1	30.9	TAPI-2	0.2	1.8	0.3
2	wtHIV-1	26.4	IgGs	0.2	6.9	0.3
2	wtHIV-1	28.7	Anti-TNFα Abs	0.2	0.2	<0.1
1	Δ*nef*HIV-1	34.7	Complete medium	0.5	1.8	0.6
1	Δ*nef*HIV-1	31.3	GW4869+ Spiroepoxide	0.3	0.9	0.3

CD4^+^ T lymphocytes were activated, infected with the indicated VSV-G psedotyped HIV-1 strains and, after 3 days, put in the upper chamber of a *trans*-well plate in the presence or not of the indicated inhibitors. As a control, activated, uninfected cells (Ctrl) were also assayed. In the third left column, shown are the percentages of HIV-1 expressing cells 3 days after infection as means of duplicates. Infected lymphocyte cultures were then put in separate *trans*-well co-cultures where in the lower chamber autologous resting CD4^+^ T lymphocytes were seeded 72 h after challenge with Δ*env* HIV-1 in the absence or presence of AZT. As a control, mock infected cells were also seeded. Percentages of HIV-1 Gag expressing cells were scored after 48 h of co-cultivation in the presence of AZT. Shown are the percentages of HIV-1 expressing cells as detected by intracytoplasmic CAp24 FACS analysis as means of duplicates of independent experiments performed with PBMCs from the number of healthy donors indicated in the first left column

The results from this set of experiments support the idea that extra-cellular factors released by HIV productively infected cells have no major influence on the exosome-dependent activation of latent HIV.

### Activation of latent HIV-1 by exosomes from cells infected with a non-producer HIV-1

**Fig. 7:**
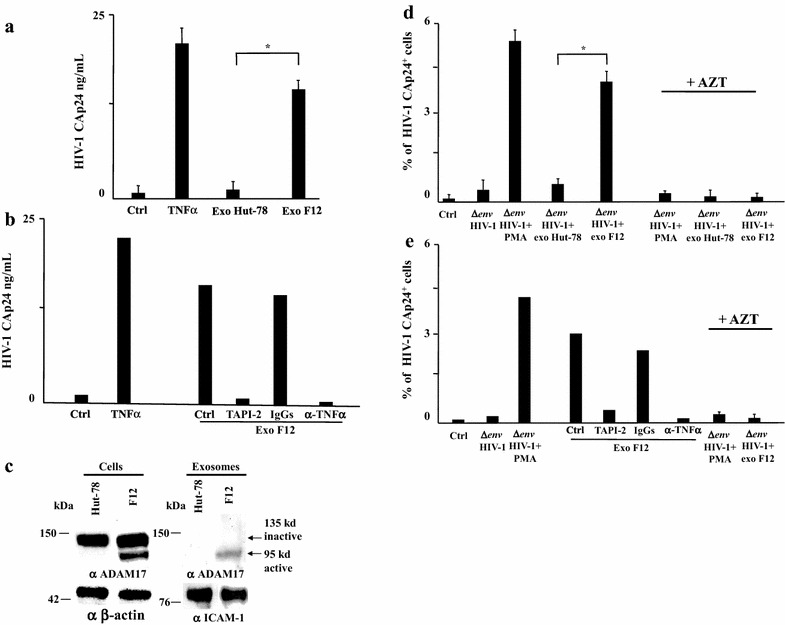
Latent HIV-1 is activated by exosomes from cells infected with defective HIV-1. **a** A total of 120 μU of exosomes purified from the supernatants of either parental Hut-78 or Hut-78/F12 cells were used to challenge 5 × 10^4^ U1 cells in U-bottom 96 well plates. After 24 h, cells were extensively washed, and released HIV-1 was measured as CAp24 concentration in cell supernatants after additional 24 h. As a control, cells were either left untreated (Ctrl) or treated with 100 ng/mL of recombinant TNFα. The results are expressed as mean values + SD from six independent experiments carried out with duplicates. *p < 0.05. **b** The same experiment whose data are reported on *panel* **a** has been reproduced on U1 treated with either TAPI-2, anti-TNFα Abs, or isotype matched IgGs soon after the exosome challenge. Shown are the results calculated as mean values from two experiments with duplicates. **c** Western blot analysis for the expression of ADAM17 in either Hut-78 or Hut-78/F12 cells. Signals from cellular ADAM17 were normalized with β-actin signals, whereas exosome preparations were also probed for the presence of ICAM-1. On the *left* of each *panel*, molecular weight markers are given in kDa. On the *right*, *arrows* identify both inactive and active ADAM17 forms. The results are representative of two independent experiments. **d** Effects of exosomes from Hut-78/F12 cells on HIV-1 latently infecting primary CD4^+^ T lymphocytes. A total of 5 × 10^4^ of untouched primary CD4^+^ T lymphocytes was challenged by spinoculation with (VSV-G) Δ*env*HIV-1 in the presence or not of AZT. As a control, conditions with unchallenged CD4^+^ T lymphocytes (Ctrl) were included. After 48 h, HIV-1 latently infected cells were extensively washed and then *left* in culture for additional 24 h. Afterwards, cells were challenged with 120 μU of exosomes from either Hut-78 or Hut-78/F12 cells. Shown are the percentages of HIV-1 Gag expressing cells as detected by intracytoplasmic CAp24 FACS analysis 24 h post-exosome challenge. As a control, cells were either treated with PMA + ionomycin (PMA), or *left* untreated (Ctrl). In addition, CD4^+^ T lymphocytes challenged with (VSV-G) Δ*env*HIV-1 in the presence of AZT were treated with either PMA + ionomycin or exosomes from either Hut-78 or Hut-78/F12 cells. Shown are the results calculated as mean percentage values + SD of triplicate cultures of CD4^+^ T lymphocytes from three donors. *p < 0.05. **e** The same experiment whose data are reported in *panel* **d** has been reproduced using CD4^+^ T lymphocytes treated with either TAPI-2, anti-TNFα Abs, or isotype matched IgGs soon after exosome challenge. The results are shown as mean percentage values from two experiments with duplicate cultures

The majority of integrated HIV-1 genomes present in cART-treated patient is defective [44–48], and, at least in part, transcriptionally active [49]. We were interested in understanding whether exosomes released by cells expressing defective HIV-1 can activate latent HIV-1. To this end, we purified exosomes from a cell line, referred to as Hut-78/F12, chronically infected with a non-producer, full length HIV-1 variant. It expresses a complete viral protein pattern comprising a truncated Vpr, an uncleaved Env gp160 [50], and a mutated Nef which retains the domain involved in the uploading of activated ADAM17 in exosomes [14]. As a control, exosomes from parental Hut-78 cells were used. The effects of exosomes from the two cell lines were analyzed in terms of activation of latent HIV-1 in both U1 cells and HIV-1 latently infected primary CD4^+^ T lymphocytes. When 120 μU of exosomes from Hut-78/F12 cells were used to challenge U1 cells, virus activation become readily detectable in terms of release of Gag protein in the supernatant. Differently, exosomes from parental Hut-78 cells proved ineffective (Fig. 7a). The effects of exosomes from Hut-78/F12 cells were abolished by treatment with either TAPI-2 or anti-TNFα Abs (Fig. 7b), in line with the detection of activated ADAM17 in exosomes from Hut-78/F12 but not Hut-78 cells (Fig. 7c).

Consistently, we observed that treatment with exosomes from Hut-78/F12 cells, but not parental Hut-78, activated latent HIV-1 infecting primary CD4^+^ T lymphocytes (Fig. 7d), and that this effect was abolished by both TAPI-2 and anti-TNFα Abs (Fig. 7e).

We concluded that latent HIV-1 can be activated by exosomes from cells infected with defective HIV-1. These effects may mirror events leading to reservoir activation possibly occurring in cART-treated patients, where a relevant part of integrated HIV-1 is both defective and transcriptionally active.

### Lack of HIV-1 replication in activated CD4^+^ T lymphocytes challenged with exosomes purified from HIV-1 infected cells

We here provide evidence that HIV-1 latently infecting CD4^+^ T lymphocytes can be activated by the treatment with exosomes from cells infected with HIV-1 expressing a functional Nef. Because HIV-1 infectivity can be enhanced by exosomes [51], we took particular care in ruling out the presence of HIV-1 infectious particles contaminating our exosome preparations which might have contributed to HIV-1 replication detected in HIV-1 latently infected cells challenged with exosomes. To assess the presence of infectious HIV-1 in the exosome preparations, 5 × 10^4^ PHA-activated primary CD4^+^ T lymphocytes (i.e., the same number of HIV-1 latently infected cells we used for exosome treatments) were challenged by spinoculation with the largest volumes of each exosome preparation we used for experiments on HIV-1 latently infected cells. As a control, cells were infected with 50 pg CAp24 equivalents of HIV-1. Twenty-four hours later, the cells were extensively washed and the cultures monitored for HIV-1 replication in terms of both CAp24 release in supernatants and intracellular CAp24 cell expression until 7 days after challenge. As shown in Table 2, no evidence of HIV-1 infection was found in all cell cultures challenged with the various exosome preparations. The rather low levels of CAp24 detected in some instances in supernatants at the earliest day post infection most likely reflected the presence of residual exosome vesicles which, as already described, can incorporate HIV-1 Gag molecules when produced by HIV-1 infected cells [9, 14]. Accordingly, similar results were observed by pre-treating target cells with AZT (not shown).

**Table 2:** Lack of HIV-1 replication in PHA-activated CD4^+^T lymphocytes challenged with different exosome preparations

Days post-challenge input	CAp24 pg/ml			% of HIV-1^+^ cells		
	2	5	7	2	5	7
Exo U937	<15	<15	<15	<1	<1	<1
Exo U937 Δ*nef* HIV-1	31	<15	<15	<1	<1	<1
Exo U937 Δ*nef* HIV-1 + wtNef	26	<15	<15	<1	<1	<1
Exo U937 Δ*nef* HIV-1 + Nef_G2A_	44	<15	<15	<1	<1	<1
Exo CD4^+^ T lymphocytes	<15	<15	<15	<1	<1	<1
Exo CD4^+^ T lymphocytes + HIV-1	<15	<15	<15	<1	<1	<1
Exo MDMs	<15	<15	<15	<1	<1	<1
Exo MDMs + HIV-1	<15	<15	<15	<1	<1	<1
Exo Hut-78	<15	<15	<15	<1	<1	<1
Exo Hut-78/F12	89	<15	<15	<1	<1	<1
HIV-1, 50 pg	<15	9980	2400	<1	32.2	7.9

A total of 5 × 10^4^ CD4^+^ T lymphocytes were spinoculated with 120 μU of the exosome preparations indicated in the left column. As a control, the same number of cells was challenged with 50 pg CAp24 equivalents of the T-tropic NL4-3 HIV-1 strain. Cell cultures were washed 24 h later, and viral replication was monitored at the indicated days post-infection by measuring CAp24 levels in supernatants as well as scoring HIV-1 expressing cells by CAp24 FACS analysis. Shown are mean values obtained by challenging cells from two healthy donors with duplicate conditions

Even though it is basically impossible to rule out the presence of minimal amounts of infectious viral particles contaminating our exosome preparations, we concluded that the HIV-1 expressing cells we detected in HIV-1 latently infected cells 24 h after exosome challenge did not originate from the infection with contaminating infectious HIV-1 particles.

## Discussion

Cells infected with either replication-competent or defective HIV-1 release exosomes uploading activated ADAM17 [14, 15]. These “armed” nanovesicles induce cell activation in target quiescent CD4^+^ T lymphocytes which then become susceptible to HIV replication. By extending the investigations to HIV-1 latent infection, we observed that exosomes from HIV-1 infected cells activate the HIV-1 latently infecting U1 cells. Virus activation relies on both uploading of activated ADAM17 in exosomes and release of TNFα from target cells. The results we obtained using HIV-1 strains deleted or mutated in *nef* are consistent with our previous findings [15], hence further enforcing the idea that the uploading of activated ADAM17 in exosomes depends on the expression of Nef in exosome-producing cells. On the other hand, we had no evidences suggesting the involvement of exosome-associated Nef in the activation of latent HIV-1.

Apparently uninfected small sub-populations were detectable within HIV-1 infected U937 cells we used as exosome source. Since it is known that the expression of Nef in exosome-producing cells is sufficient for the uploading of activated ADAM17 in exosomes [15], the presence of these cells lacking HIV-1 expression but actually expressing Nef was not expected to influence the downstream results.

Many protocols to recover HIV-1 latently infected cells have been developed and recently compared in a quite comprehensive study [38]. We developed a system basically similar to that described by Greene’s group [31]. Key experimental points consist in leaving CD4^+^ T lymphocytes untouched during the immunomagnetic selection from PBMCs, and challenging them with (VSV-G) Δ*env* HIV-1. The use of this viral strain guaranteed optimal levels of viral entry in target cells meanwhile avoiding, when latent HIV-1 is activated, re-infection events potentially biasing the interpretation of results. The overall efficiency of infection we detected after cell activation (3–6 % of HIV-1 expressing cells) appeared fully consistent with that previously reported using different HIV-1 derivatives [2, 31].

In a quite similar system of HIV-1 latency, exogenous TNFα was shown to activate latent HIV-1 with a very low efficiency [31]. Consistently, in the here described *trans*-well co-cultures where activated donor lymphocytes were expected to release TNFα, no apparent activation of latent HIV-1 occurred in the presence of inhibitors of exosome release. Most likely, additional molecular events besides the release of TNFα from target cells are necessary for the activation of latent HIV-1. Exact delineation of the mechanisms underlying the exosome-induced activation of HIV latency requires additional investigations.

We can exclude that the activation of latent HIV-1 we observed in the different cell/exosome systems used depended on the presence of infectious HIV-1 particles contaminating the exosome preparations. In fact, when cells most susceptible to HIV infection, i.e., activated primary CD4^+^ T lymphocytes, were challenged with our exosome preparations, no viral replication was detectable. These data were obtained in experimental conditions much more stringent than those used in the HIV-1 latency assays, i.e., using activated rather than resting CD4^+^ T lymphocyte target cells, and prolonging the observation period to 7 days instead of the 24 h incubation we considered after exosome treatment.

The high mutation rate occurring in HIV-1 retrotranscription leads to the emergence of defective HIV-1 genomes which can persist in blood cells of HIV-1 infected patients. These cells are expected to have a longer life span than cells infected with replication-competent quasispecies as a consequence of the attenuated cytotoxicity of mutated viral products [45]. The presence of a relevant burden of defective HIV-1 genomes integrated in PBMCs of infected patients was demonstrated in both PBMCs and rectal tissues of cART-treated patients [46]. It was estimated that the percentage of defective HIV-1 genomes in viral reservoirs of treated patients overrides that of replication-competent ones by more than 100-folds [48]. This hypothesis was also supported by the evidence that treatment of HIV-1 reservoirs with various cell activation factors invariably led to virus release from only a minority of cells integrating HIV-1 [47]. Hence, investigating whether activation of latent HIV-1 can also occur when exosome-producing cells are infected with defective HIV-1 is of relevance. Here, we provide evidence that exosomes released by cells infected with defective HIV-1 can induce activation of latent HIV-1. In infected patients, any defective but transcriptionally active HIV strain would be part of this mechanism whatever their mutations/deletions, except those inactivating either Tat or the Nef domain involved in the uploading of ADAM17 in exosomes.

Whether or not cART totally halts virus replication is still under debate. In the case of a complete block of HIV-1 replication, exosome-dependent reactivation of latent HIV-1 would lead to a progressive depletion of the latent reservoir due to both cell death of virus-expressing cells and immune surveillance. In contrast, when cART leakiness occurs to some extent, exosomes from cells infected with defective HIV-1 strains (insensitive to cART) may contribute to the re-activation of the latent reservoir leading to the episodes of transient viremia (“blips”) occurring in cART treated patients [52–55].

## Conclusions

The presence of latent reservoirs is a major hurdle towards HIV eradication. Hence, the study of mechanisms underlying activation and transmission of latent HIV infection is relevant not only to clarify still unknown aspects of HIV pathogenesis, but also to improve the therapeutic approaches aimed at virus eradication. Here, we provide evidence that latent HIV-1 can be activated by exosomes released by HIV-1 infected cells. This event is mediated by the activity of ADAM17 uploaded in exosomes and the consequent release of TNFα from target cells.

## Methods

### Cell cultures and isolation

U1, U937 cells and derivatives thereof, Hut-78, Hut-78/F12 [50] were grown in RPMI medium plus 10 % heat-inactivated fetal calf serum (FCS). Human embryonic kidney 293T cells were grown in Dulbecco’s modified Eagle’s medium plus 10 % heat-inactivated fetal calf serum (FCS). CD4^+^ T lymphocytes were isolated from PBMCs of healthy donors by negative selection using an immunomagnetic-based kit (Miltenyi), and cultivated in RPMI medium plus 10 % FCS. Cell cultures were checked for purity through FACS analysis for CD4, CD8, and CD14 markers. Cell preparations having more than 3 % of CD8^+^ cells and/or 1 % of CD14^+^ cells were discarded. For cell activation, either 10 ng/mL of PMA plus 0.5 μg/mL of ionomycin or 2 μg/mL of PHA were added to CD4^+^ T lymphocyte cultures. Monocytes were isolated from PBMC of healthy donors using an immunomagnetic monocyte selection kit (Miltenyi). Purity of recovered cell populations was assayed by FACS analysis using PE-conjugated anti-CD14 mAb (Becton–Dickinson). To obtain human primary monocyte-derived macrophages (MDMs), monocytes were cultured for 7 days in 48 well plates in RPMI supplemented with 20 % FCS.

For Nef activation, HT from Sigma was solubilized in DMSO and used at 50 nM. TAPI-2 was purchased from Santa Cruz Biotechnology and used at 1 μM. Recombinant human TNFα was bought from R&D Systems. AZT was obtained from NIH AIDS Research and Reference Reagent Program, and used at 10 μM. For anti-TNFα neutralization experiments, either anti-TNFα neutralizing antibodies (polyclonal rabbit antibodies, Fitzgerald Industries) or normal rabbit IgGs were added to CD4^+^ T lymphocytes immediately after exosome challenge. The same amounts of antibodies were then re-added after 24 h of culture.

### Molecular clones, transfections, and HIV-1 infections

Preparations of VSV-G pseudotyped HIV-1 were obtained from supernatants of 293T cells 48 h after transfection with either pNL4-3 HIV-1 molecular clone or its Δ*env* and Δ*nef* derivatives co-transfected with a pcDNA3.1 (Invitrogen)-based vector expressing VSV-G in 5:1 molar ratio. Transfections were performed using Lipofectamine 2000 (Invitrogen). Supernatants were clarified and concentrated by ultracentrifugation on a 20 % sucrose cushion as previously described [56]. This method ensured that exosomes from transfected cells were excluded from the vesicle pellet [57]. Virus preparations were titrated in terms of HIV-1 CAp24 content using quantitative enzyme-linked immunosorbent assay (ELISA, Innogenetic). Infections with HIV-1 were carried out by spinoculation at 400×*g* for 30 min at room temperature (r.t.) using 500 ng CAp24 equivalents of HIV-1 for 10^6^ cells.

### Establishment of CD4^+^ T lymphocyte cultures latently infected with HIV-1

Untouched CD4^+^ T lymphocytes isolated from PBMCs of healthy donors were spinoculated using 300 ng CAp24 equivalents of (VSV-G) Δ*env* HIV-1/10^6^ cells in the presence or not of AZT. After 48 h, cell cultures were extensively washed and refed. One day later, CD4^+^ T lymphocytes were analyzed for intracytoplasmic contents of HIV-1 Gag products. Cell cultures with more than 0.3 % of positive cells for HIV-1 Gag expression were discarded. Otherwise, CD4^+^ T lymphocytes were challenged with exosomes or put in *trans*-well co-cultures, and HIV-1 expression was evaluated after additional 24-48 h.

### *Trans*-well co-cultures

*Trans*-well co-cultures were carried out in 12-well plates using Cell Culture Insert Falcon Membrane (25 mm diameter, 0.4 μm pore size, Becton–Dickinson). For preparation of exosome-donor cells, CD4^+^ T lymphocytes were activated for 48 h, and then infected with spinoculation with 200 ng CAp24 equivalents of (VSV-G) wtHIV-1/10^6^ cells or equivalent amounts of the Δ*nef* derivative. After additional 72 h, the percentage of HIV-1 infected cells was evaluated by Gag-specific FACS analysis. In the presence of at least 25 % of infected cells, cell cultures were seeded in the upper chamber of *trans*-well plates while HIV-1 latently infected autologous CD4^+^ T lymphocytes obtained as described above were seeded in the bottom chamber. AZT was then added to the co-cultures which were run for 48 h in the presence or not of 1 μM of either GW4869 (Sigma) and spiroepoxide (Santa Cruz) inhibitors of exosome synthesis [39–43], TAPI-2 [30], or anti-TNFα Abs. Thereafter, target lymphocytes seeded in the bottom chambers were analyzed by FACS for the expression of intracytoplasmic HIV-1 CAp24.

### Nanovesicle purification and challenge

Cell culture supernatants containing exosome-depleted FCS were processed following already described methods for exosome purification [58, 59]. In detail, supernatants were centrifuged at 500×*g* for 10 min. Then, the supernatants underwent differential centrifugations consisting in a first ultracentrifugation at 10,000×*g* for 30 min. Supernatants were then harvested, filtered with 0.2 μM pore size, and ultracentrifuged at 70,000×*g* for l h. The pelleted vesicles were washed in 1 × PBS, and ultracentrifuged again at 70,000×*g* for 1 h. Afterwards, the pellet was resuspended in 200–400 µL of 1 × PBS and subjected to discontinuous iodixanol (Axis-Shield) gradient. Briefly, concentrated vesicles were ultracentrifuged at 200,000×*g* for 1.5 h at 4 °C in an SW41 Ti rotor (Beckman) through a 6–18 % iodixanol density gradient formed by layering iodixanol in 1.2 % increments. Then, 0.7 mL fractions were collected starting from the top. In some instances, half of each fraction was diluted with 2 volumes of 0.9 % sodium chloride and ultracentrifuged for 30 min at 95,000 rpm in a TL-100 tabletop ultracentrifuge. Finally, the pellet was resuspended in 50 µL of Tris–HCl pH 7.4 10 mM, NaCl 100 mM, EDTA 1 mM. A total of 5 × 10^4^ cells/condition was used for exosome challenges which were performed by spinoculation as described for HIV-1 infection.

### Detection of AchE, ADAM17 and TNFα contents in exosomes

Vesicle-associated AchE activity was evaluated through Amplex Red kit (Molecular Probes) following the manufacturer’s recommendations. The AchE activity was measured as mU/mL, where 1 mU is defined as the amount of enzyme which hydrolyzes 1 pmol of acetylcholine to choline and acetate per minute at pH 8.0 at 37 °C. The activity of exosome-associated ADAM17 was assayed through the Innozyme TACE activity kit (Calbiochem) which was carried out on 1 mU equivalent of AchE activity of each exosome preparation following the manufacturer’s recommendations. Measurement of TNFα in the exosome preparations was performed through ELISA kit from Immunological Sciences following the manufacturer’s recommendations.

### FACS analysis of cells and nanovesicles

To detect intracytoplasmic HIV-1 CAp24, cells were treated with trypsin for 15 min at 37 °C. Then, they were labeled using the KC57-RD anti-CAp24 monoclonal antibody (Coulter) upon permeabilization with Cytofix/Cytoperm solutions (BD Pharmingen) as previously described [56]. HIV-1 Gag and Nef double staining was performed by permeabilizing cells in a similar way, and then incubating them with the anti-Nef MATG mAb (generous gift of O. Schwartz, Institute Pasteur, Paris, France). Cells were then labeled with FITC-conjugated anti-mouse IgG Abs, and finally with the KC57-RD anti-CAp24 mAb.

Double staining of nanovesicles was performed by incubating them with 5 µL of surfactant-free white aldehyde/sulfate latex beads overnight at r.t. on a rotating plate. Afterwards, nanovesicle-bead complexes were washed and incubated at 37 °C for 2 h with FITC-conjugated cholera toxin, subunit B (CTX-B) for GM1 detection. Then, the samples were washed and incubated with PE-conjugated anti-CD63 monoclonal antibody (BD Pharmingen) 1 h at 37 °C. Finally, the beads were washed, resuspended in 1 × PBS-2 % v/v formaldehyde, and FACS analyzed.

### Western blot assay

Western blot analysis on cell lysates was performed by washing cells twice with 1 × PBS (pH 7.4) and lysing them for 20 min on ice with lysis buffer (20 mM HEPES pH 7.9, 50 mM NaCl, 10 mM EDTA, 2 mM EGTA, 0.5 % nonionic detergent IGEPAL CA-630, 0.5 mM dithiothreitol, 20 mM sodium molybdate, 10 mM sodium orthovanadate, 100 mM sodium fluoride, 10 μg/mL leupeptin, 0.5 mM phenylmethylsulfonyl fluoride). Whole cell lysates were centrifuged at 6000×*g* for 10 min at 4 °C. Protein concentration of cell extracts was determined by the Lowry protein quantitation assay. Aliquots of cell extracts containing 30–50 μg of total proteins were resolved by 12 % sodium dodecyl sulfate–polyacrylamide gel electrophoresis (SDS-PAGE) and transferred by electroblotting on a 0.45 μm pore size nitrocellulose membrane (Amersham) overnight using a Bio-Rad Trans-Blot. Antibodies used in immunoblots were: pools of strongly HIV-1 positive human sera, sheep polyclonal anti-Nef ARP444 (a generous gift from Dr. Mark Harris), rabbit polyclonal anti-ADAM17 from Cell Signaling, and monoclonal anti-β-actin AC-74 (Sigma). Immune complexes were detected with horseradish peroxidase-conjugated secondary antibodies (GE Healthcare) and enhanced chemiluminescence reaction (Euroclone).

### Statistical analysis

When appropriate, data are presented as mean + standard deviation (SD). In some instances, the paired Student’s *t* Test was used and confirmed using the non-parametric Wilcoxon rank sum test. *P* < 0.05 was considered significant.
